# Communication coaching for sonographers (CCS): Preliminary evaluation of a novel intervention to improve unexpected news delivery

**DOI:** 10.1016/j.pecinn.2023.100231

**Published:** 2023-10-31

**Authors:** Judith Johnson, Essie Kaur, Jane Arezina, Louise D. Bryant, Rebecca Wallace, Gill Harrison, Ruth Bender Atik, Jen Coates, Natasha Hardicre, Roxanne Sicklen, Teresa Lardner, Karen Horwood, Hannah Beety, Jon Arnold, Kathryn I. Pollak

**Affiliations:** a School of Psychology, University of Leeds, Leeds LS29JZ, UK; b Bradford Institute for Health Research, Bradford BD9 6RJ, UK; c University of New South Wales, Sydney, NSW 2052, Australia; d Health Services Management Centre, University of Birmingham, 40 Edgbaston Park Road, Birmingham B15 2TT, UK; e Specialist Science Education Department (SSED), Leeds Institute of Cardiovascular and Metabolic Medicine, School of Medicine, Worsley Building, University of Leeds, LS2 9JT, UK; f Faculty of Medicine and Health, University of Leeds, LS29JT, UK; g Aneurin Bevan University Health Board, Royal Gwent Hospital, Cardiff Road, Newport NP20 2UB, UK; h The Society and College of Radiographers, 207 Providence Square, Mill Street, London SE1 2EW, UK; i City, University of London, Northampton Square, London EC1V 0HB, UK; j The Miscarriage Association, 2 Otters Holt, Wakefield WF4 3QE, UK; k Sands, 10–18 Union Street, London SE1 1SZ, UK; l NHS England, Quarry House, Leeds LS27UE, UK; m Royal Free London NHS Foundation Trust, Barnet Hospital, Wellhouse Lane, Barnet, Herts EN5 3DJ, UK; n Public Health Commissioning and Operations, Fetal Anomaly Screening Programme, NHS England, UK; o Independent Lay Expert, Leeds, West Yorkshire, UK; p Nest Independent Midwifery, Kirkstall, Leeds, UK; q Tiny Tickers, PO Box 369, Leeds LS26 1FR, UK; r Duke University School of Medicine and Duke Cancer Institute, Durham, NC 27705, USA

**Keywords:** Communication, Coaching, Breaking bad news, Pregnancy, Ultrasound, Burnout

## Abstract

**Objective:**

Obstetric ultrasound scans provide real-time results. In some organisations and countries, the immediate communication of results by sonographers to patients is standard practice, but there is a lack of evidence-based training to support them with this challenging task. This pilot study evaluated a novel communication coaching intervention to improve sonographer communication.

**Methods:**

Coaches met with sonographers(*N* = 15) three times. Sonographers collected three audio recordings of scans involving unexpected news communication at baseline(R1), post-Session 1(R2) and post-Session 2(R3), which were rated for communication skills. Participants self-reported communication confidence and burnout before(T1) and after(T2) the intervention. Feedback was collected at T2. Data were analysed using paired-samples *t*-tests with bootstrapped significance estimates.

**Results:**

*N* = 10 sonographers completed the intervention. There were significant increases in communication skills(R1 m = 4.85, SD = 1.07; R3 m = 6.73, SD = 1.80, *p* = 0.003) and communication confidence(T1 m = 28.00, SD = 6.27; T2 m = 32.80, SD = 6.05, *p* = 0.005). There were no significant changes in burnout(*p* > 0.05). All respondents said they would recommend the intervention and most strongly agreed it was engaging(*n* = 8; 89%) and imparted useful skills(n = 8; 89%).

**Conclusion:**

Communication coaching is an acceptable, potentially effective tool for improving communication of unexpected news by sonographers in ultrasound.

**Innovation:**

This is the first evaluation of an intervention to support obstetric sonographers with news delivery.

## Highlights

• We evaluated Communication Coaching for Sonographers (CCS).
• CCS was associated with significant improvements in unexpected news delivery.
• CCS was associated with significant improvements in communication confidence.
• CCS was not associated with any changes in burnout.
• CCS was not associated with any changes in the length of scans in minutes/S.

## 1 Introduction

At least 15% of pregnancies result in pregnancy or baby loss [1], and in 2%, a potential congenital health condition is identified [2]. Ultrasound is a key diagnostic tool in obstetrics, which generates results for the sonographer in real-time. In the current paper, we will use the term ‘sonographer’ to describe all qualified practitioners who conduct obstetric ultrasound scans. Immediate communication of ultrasound results is consistent with patients' preferences [3,4] and so countries and organisations are increasingly adopting this approach [[5], [6], [7]]. In the UK, sonographers have been communicating directly with patients for 20 years, and this practice is endorsed in both UK guidance for sonographers [7] and national policy recommendations [8].

While immediate communication of results is in line with patient preferences, this practice can be challenging for sonographers, particularly when findings are uncertain or unexpected [9]. In the current report ‘unexpected news’ will be used in place of ‘bad news’, as this is the established preferred term when referring to pregnancy findings [10]. Sonographers do not know what they will find prior to commencing the scan; as they perform the scan, patients often watch their face for ‘clues’, especially if they are anxious about their pregnancy [3,11]. As such, sonographers find themselves carefully managing their body language and facial expressions, in addition to conducting and formulating the scan results [11]. They are then required to decide which words and phrases they will use to share this information with patients with no time to prepare for the conversation. Unsurprisingly, many studies have reported that patients can have negative experiences of receiving unexpected news following ultrasound, describing sonographers who have used inconsiderate words or phrases, or who have seemed unable to clearly state what was found [3,4,12]. These experiences can be devastating for patients, compounding the distress that may be intrinsic to the news of the finding itself [12,13].

There are established news delivery frameworks developed in other healthcare settings [14,15], but the uniquely challenging aspects of communication in ultrasound mean that these do not translate easily to obstetric scanning. First, these guidelines instruct providers to spend time preparing the words they will use to communicate their findings, time which sonographers do not have; second, they do not recognise the need for balanced and neutral language, which is a cornerstone of good communication in obstetric ultrasound [6,8,10]. To address these gaps, we developed a framework using a consensus approach. The framework is called ASCKS (Avoid assumptions; Set up the scan; Clear, honest information; Kindness; Self-care) [10] (Table 1). The ASCKS guidelines make specific phrasing suggestions to support sonographers to communicate honestly, using sensitive, neutral and clear terms [10].

**Table 1:** Items based on the ASCKS framework which were used to generate the 12-item observer communication skills rating scale.

	Item	Item description
Avoid assumptions and loaded words	Uses neutral language throughout	Does not use the terms ‘wrong’ or ‘normal/abnormal’ (unless using it to reassure)
Set up the scan	Asks about feelings about the pregnancy	Asks a question similar to, ‘How are you feeling about the pregnancy?’ prior to the scan.
	Prepares patient for the possibility of an internal scan	Informs patient prior to the first scan that an internal scan may be needed following the trans-abdominal scan, or establishes that an internal scan will not be needed.
	Explains silence	Informs patient prior to the scan that they will be silent for a while during the scan.
	Explains use of the monitor	Informs patient prior to the scan of how and when they will offer to share the screen/images with them.
Clear, honest information	Seeks permission to communicate findings using the monitor	After the scan has been conducted, the sonographer offers to show the monitor to the patient.
	Communicates findings using widely understood terms	When communicating the findings, the sonographer uses the word ‘miscarriage’ if indicated, or other suitable and widely understood term
Kindness	Expresses regret	When delivering the news, the sonographer uses the term ‘sorry’ and/or ‘unfortunately’ and/or ‘I'm afraid’
	Emotion naming	Names an emotion on one or more occasions (e.g., ‘surprise’; ‘anxiety’; ‘devastating’; ‘upsetting’; ‘tense’). This shouldn't be framed as an instruction, e.g., ‘Don't worry’, or future framed e.g., ‘If you're feeling worried about it’.
	Emotional experience naming	Names an emotional experience on one or more occasions, e.g., ‘uncomfortable’; ‘stressful’; ‘awful’; ‘hard’; ‘difficult’; ‘not nice’. This shouldn't refer to a physical experience, e.g., ‘that could be sore’, or to a choice, e.g., ‘if you're comfortable with that?’
	Wish statement	Uses a wish statement, e.g., ‘I wish I had different news for you’
	Use of ‘baby’	The term ‘baby’ is used as default, unless patient uses other term, e.g., ‘fetus’

The ASCKS framework is endorsed by UK professional guidelines [16] and policy [8], and has influenced similar Australian guidelines [6]. There is now a need to understand how sonographers can be supported to communicate using these guidelines in day-to-day clinical practice. While sonographers often receive some education in news delivery as part of their pre-qualification training [9] and can choose to attend training days post-qualification [17], effectiveness of this training in improving communication skills has not been demonstrated [15]. Furthermore, feedback suggests that existing training is often reliant on didactic lectures and that it could be improved by reducing reliance on didactic teaching and increasing use of feedback on real clinical encounters [10]. There is an urgent need to develop and evaluate effective training interventions in this group.

The current study addresses this by conducting a preliminary evaluation of a tailored communication coaching intervention for sonographers. Communication Coaching is a one-to-one, supportive intervention, where a communication coach provides encouraging feedback based on providers' own recorded and transcribed patient encounters [18]. This has been evaluated in a range of healthcare professional groups including physicians, physician assistants, and nurses [19,20], to improve both generic consultation communication styles and specific forms of provider-patient communication, such as goals of care conversations [18,21].

To ensure relevance of the intervention for obstetric sonographers, a previous study investigated how Communication Coaching could be adapted to support sonographers to deliver unexpected pregnancy news [22]. This study recruited a range of stakeholders, including sonographers and former patients with lived experience and found that all groups thought the intervention was feasible with small practical adaptations to the coaching sessions. These included a stronger emphasis on flexible timing and delivery via an online platform (i.e., Teams/Zoom), both of which were incorporated into the intervention plan [22]. There is now a need to evaluate the adapted intervention, Communication Coaching for Sonographers (CCS) in practice, to explore whether it is acceptable to sonographers and could have the potential to be effective. As such, the present study aimed to conduct a preliminary evaluation of CCS in pregnancy ultrasound.

## 2 Methods

### 2.1 Design

We conducted a single-arm study with a before-after design evaluating CCS. Outcome variables were guided by the Kirkpatrick model [23], which recommends evaluating interventions on four levels: 1) reactions and views of participants; 2) knowledge and skills; 3) behavioural changes (whether learning is applied); 4) results (whether there are changes within the organisation).

For Level 1, we collected feedback regarding participants' views of the training. For Levels 2 and 3, we collected audio recordings of scan appointments at three time points and rated these for observed news delivery skills. For Level 4, we collected data relating to communication confidence, burnout and turnover intentions. We also collected data regarding the length of scan appointments, to establish whether there were any increases in appointment time following the coaching.

### 2.2 Eligibility and setting

Participants were eligible for the study if they 1) were qualified sonographers or ultrasound practitioners, 2) were scanning in obstetrics, 3) had some sessions within the Early Pregnancy Unit (EPU) of participating National Health Service (NHS) sites within the UK. We focused on collecting recordings in the EPU rather than in the routine ultrasound clinics due to an overall higher rate of unexpected findings in this setting. This approach reduced the number of recordings which would need to be collected before a scan involving unexpected news delivery would be gained. Sonographers requested permission to record from patients during all their scans but only saved and used audio recordings from patients where they had delivered unexpected news (defined as news or possible news of a pregnancy deviating from typically expected healthy development) using a Dictaphone. These were transferred securely to the research team who transcribed them. Coaching sessions were then conducted using video platform (Zoom or Teams).

### 2.3 Intervention

Communication Coaching uses a 1:1 supportive coaching format involving the review and discussion of healthcare professionals' own audio-recorded, transcribed consultations to improve their personal communication skillset [24]. It draws on adult learning principles that reinforce behaviours done correctly, suggesting “tweaks” when communication could be improved [25]. As sessions are brief and individualised, it can be delivered to fit with healthcare professionals' busy work schedules. In the present study, the intervention, CCS, involved a 30-min introduction and two subsequent 30-min coaching sessions.

### 2.4 Coach training

CCS was delivered by JJ, a licensed Clinical Psychologist who was coached in the intervention through 1) two experiential role-play sessions with a Communication Coach (KP), where she role-played a sonographer and was coached based on a sonographer's transcript, and 2) written and verbal feedback from KP on three Coaching sessions she delivered.

### 2.5 Recruitment and ethics

Sonographers within participating hospitals were approached by their manager or the local collaborator, recruited and participated between April 2022 and June 2023. The research team then liaised with interested sonographers to provide information and gather written informed consent. The study was approved by the School of Psychology, University of Leeds Ethics Committee (Ref - PSYC-601) and the UK Health Research Authority (Project ID – 303,633). As advised by an NHS Ethics Committee, patients receiving scans were not considered participants in the research and were not, therefore required to provide written informed consent. Instead, departments provided written information to patients on arrival for their scan appointments and sonographers sought verbal permission from patients at the start of appointments, prior to recording.

### 2.6 Procedure and measures

Self-report measures were completed online on Qualtrics. Sonographers responded to measures of confidence in communicating, burnout and turnover intentions before (Time 1; T1) and after (Time 2; T2) the coaching intervention. They provided three audio recordings: at baseline (Recording 1; R1), following the introduction session (Recording 2; R2) and after the second coaching session (Recording 3; R3). Sonographers provided feedback regarding the intervention at T2 to the Research Assistant (EK).

#### 2.6.1 Observer-rated news delivery skills

Transcripts were scored for the presence of 12 communication behaviours based on the ASCKS framework (Table 1). Items were scored as ‘1’ (present) or ‘0’ (absent). These were summed to create a total score at each time point. We also summed the total occurrences of ‘emotion naming statements’ and ‘emotional experience naming statements’, to create a second outcome of ‘emotion words’, consistent with previous Communication Coaching studies [18,25]. All transcripts were independently scored by two authors (JJ, EK), with differences resolved through discussion.

#### 2.6.2 Confidence in communicating

Participants were asked to rate their confidence in their ability to deliver unexpected news on a scale from 1 (‘not confident’) to 10 (‘very confident’), in four contexts: early pregnancy loss, late pregnancy loss/intra-uterine death, unexpected physical condition identified before 12 weeks and unexpected physical condition identified after 12 weeks. As there is no standard, accepted measure for assessing practitioner confidence in communicating news to patients, this approach has been used in previous similar studies [15]. We report outcomes for each item separately and the total score for the summed four items.

#### 2.6.3 Burnout

Participants responded to the widely-used, 16-item Oldenburg Burnout Inventory (OLBI) [26] which comprises two scales of exhaustion and disengagement. Items are rated from 1 (strongly disagree) to 4 (strongly agree) and include ‘during my work, I often feel emotionally drained’. The OLBI is suitable for use in healthcare professionals and has been found to have acceptable test-retest reliability over four months (*r* = 0.51, *p* < 0.001, and *r* = 0.34, *p* < 0.01, for exhaustion and disengagement respectively) and internal consistency (0.74–0.87) in previous studies [26,27].

#### 2.6.4 Turnover intentions

We measured intention to leave the hospital of employment using a single item, asking participants to select one of four options: 1) No I do not intend to leave; 2) Yes I intend to leave for another hospital/trust; 3) Yes I intend to leave for a job outside of healthcare; 4) Yes for another reason (details of the reason are then requested). As there is no standard or widely used measure for capturing turnover intentions, this approach is similar to previous studies [28].

#### 2.6.5 Scan length

Scan length was estimated via 1) audio recording time and 2) transcript word length, to assess whether there were any increases following the intervention. This dual approach was taken as length can vary due to other factors such as delays during the scan for clothes changes and bathroom visits.

#### 2.6.6 Intervention feedback

Four items were used which have been used in previous evaluations of training interventions in healthcare professionals [29,30]. One item, (‘overall, would you recommend this intervention to others?’) was scored as ‘yes’, ‘no’ or ‘maybe’. Three items (‘I found the coaching engaging’; ‘I learned skills during the coaching which will be useful in future’ and ‘there was adequate time to cover the material’) were scored on a 5-point scale from 1 (‘strongly disagree’) to 5 (‘strongly agree’).

### 2.7 Data analysis plan

Descriptive statistics were conducted and reported for all outcome variables. To determine whether there were significant differences after baseline in observer-rated news delivery skills, communication confidence, burnout and scan length, paired-samples *t*-tests were conducted, with bootstrapped estimates (5000 bootstrap samples; 95% confidence interval) [31]. Bias-corrected bootstrap confidence intervals were used [32]. Bootstrapping is a powerful, non-parametric approach which can be used when distributions do not conform to the requirements of parametric tests, such as normality, and is suitable for use in small sample sizes [31]. As turnover intentions was a nominal variable, statistical analysis was not possible and instead descriptive statistics were reported to describe any changes.

## 3 Results

### 3.1 Participant characteristics

15 participants (14 women, mean age = 38.36; SD = 5.06, age and gender data missing for 1 participant) consented to the study and provided baseline data. 12 Participants were White or White British and 3 were Asian or Asian British. Participants held the roles of sonographer (*n* = 10, including one with specialist obstetric interest), senior sonographer (n = 1), advance practitioner sonographer (*n* = 2), and radiologist (n = 1), with data missing for one participant. Participants had been employed for a mea*n* = 11.14 years (SD = 5.70; data missing for one participant). 14 participants were radiographers by background, with the remaining participant from a medical background.

Of the 15 participants, 13 received at least one coaching session and 10 (67%) completed the full intervention and completed follow-up self-report questions. Of the 13 who attended at least one coaching session, the retention rate was 77%. Full recording datasets (R1, R2, R3) were available for 11 participants, as one participant collected their final recording but was not able to attend their concluding coaching session. Three participants provided reasons for non-completion, and all of these related to personal or health problems which led to extended periods of absence from work. For descriptive data regarding outcome variables, please see Table 2.

**Table 2:** Descriptive statistics for all outcome measures.

Measure	Timepoint	Mean	Standard Deviation	Min, Max scores	Possible scale range
Communication skills	R1 (*n* = 13)	4.85	1.07	3, 7	0, 12
	R2 (*n* = 12)	4.25	1.66	2, 8	0, 12
	R3 (n = 11)	6.73	1.80	5, 10	0, 12
Emotion naming	R1 (n = 13)	0.69	0.95	0, 3	0, NA
	R2 (n = 12)	0.25	0.62	0, 2	0, NA
	R3 (n = 11)	1.64	1.50	0, 4	0, NA
Confidence (total)	T1 (n = 15)	28.00	6.27	16, 40	0, 40
	T2 (n = 10)	32.80	6.05	20, 40	0, 40
Confidence (early loss)	T1 (n = 15)	7.80	1.21	5, 10	0, 10
	T2 (n = 10)	9.00	1.05	7, 10	0, 10
Confidence (late loss)	T1 (n = 15)	6.13	2.48	2, 10	0, 10
	T2 (n = 10)	7.90	2.33	3, 10	0, 10
Confidence (<12 weeks unexpected physical condition)	T1 (n = 15)	7.07	1.67	4, 10	0, 10
	T2 (n = 10)	7.80	2.04	4, 10	0, 10
Confidence (>12 weeks unexpected physical condition)	T1 (n = 15)	7.00	1.85	3, 10	0, 10
	T2 (n = 10)	8.10	1.52	5, 10	0, 10
Burnout (exhaustion)	T1 (n = 15)	18.73	3.90	13, 25	8, 32
	T2 (n = 10)	20.30	3.30	15, 25	8, 32
Burnout (disengagement)	T1 (n = 15)	17.13	2.59	13, 21	8, 32
	T2 (n = 10)	18.00	3.06	13, 23	8, 32
Scan length (seconds(minutes))	R1 (n = 13)	832.08 (14 m)	386.72 (6 m)	371.00 (6 m), 1472.00 (25 m)	NA, NA
	R2 (n = 12)	837.42 (14 m)	329.56 (6 m)	400.00 (7 m), 1387.00 (23 m)	NA, NA
	R3 (n = 11)	733.55 (12 m)	355.69 (6 m)	353.00 (6 m), 1266.00 (21 m)	NA, NA
Scan length (word count)	R1 (n = 13)	1650.31	831.18	464.00, 3093.00	NA, NA
	R2 (n = 12)	1554.50	630.25	603.00, 2549.00	NA, NA
	R3 (n = 11)	1388.27	637.57	737.00, 2757.00	NA, NA

NA = Not applicable; there is no predefined minimum and/or maximum possible score.

### 3.2 Observer rated communication skills

**Fig. 1:**
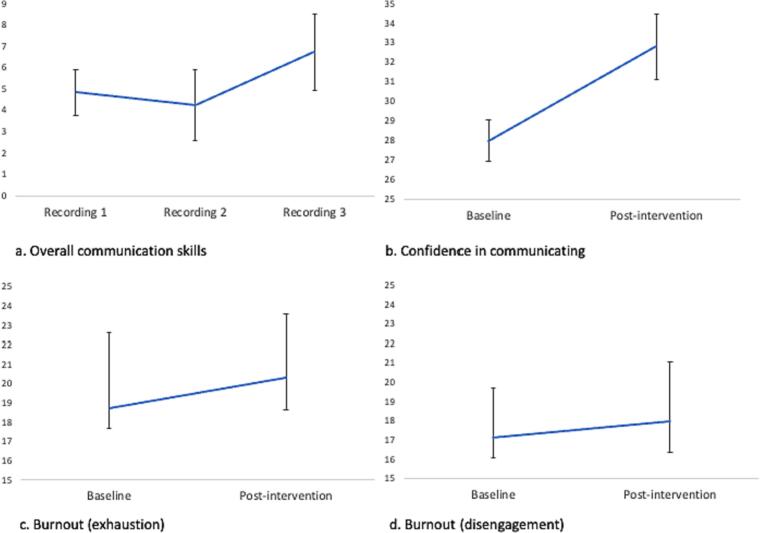
Scores for (a) Observer-rated news delivery according to the 12-point ASCKS framework scale; (b) confidence in communicating unexpected news (total); (c) exhaustion subscale of the burnout inventory; (d) disengagement subscale of the burnout inventory.

Overall communication skills increased at R3 compared with R1 (t(10) = −4.690, CI: −2.73, −1.27, *p* = 0.003; Fig. 1). There was no significant difference between emotion word scores at R3 compared with R1 (t(10) = −1.618, CI: −2.09, 0.18, *p* = 0.130).

### 3.3 Confidence in communicating

There was a significant increase in communication confidence overall at T3 compared with T1 (t(9) = −4.392, CI:-8.4, −3.7, *p* = 0.005) (Fig. 1). When items were considered individually, there were significant increases in communication confidence regarding early pregnancy loss (t(9) = −4.993, CI:-1.7, −0.9, *p* = 0.007), late pregnancy loss/intra-uterine death (t(9) = −3.139, CI:-3.05, −0.60, *p* = 0.017), unexpected physical condition identified before 12 weeks (t(9) = −2.623, CI: −2.20, −0.50, *p* = 0.040) and unexpected physical condition identified after 12 weeks (t(9) = −4.707, CI:-2.10, −1.20, *p* = 0.044).

### 3.4 Burnout

There were no significant differences in exhaustion (t(9) = −1.037, CI:-2.00, 0.400, *p* = 0.306) or disengagement (t(9) = −0.580, CI: −1.10, 0.50, *p* = 0.558) at T3 compared with T1 (Fig. 1).

### 3.5 Turnover

At T1 all participants (*n* = 15; 100%) stated they did not intend to leave their job. At T3, *n* = 9 (90%) respondents said they did not intend to leave their job, and *n* = 1 (10%) stated they intended to leave for another hospital/trust.

### 3.6 Scan length

There was no significant difference in scan length as measured by total time (t(10) = 1.490, CI: −47.20, 368.29, 368.29, *p* = 0.165) or total words (t(10) = 1.79, CI: 34.49, 784.94, *p* = 0.126) between T1 and T3.

### 3.7 Intervention feedback

Nine participants provided intervention feedback data. All respondents agreed that they would recommend CCS to others (*n* = 8; 100%; data missing for 1 participant). *N* = 8 (89%) strongly agreed that they found CCS engaging and *n* = 1 (11%) neither agreed nor disagreed with this statement. N = 8 (89%) strongly agreed that they learned skills during CCS which will be useful in future and n = 1 (11%) disagreed with this statement. *N* = 5 (56%) strongly agreed that there was enough time to cover the material and *n* = 4 (44%) agreed with this statement.

## 4 Discussion and conclusion

### 4.1 Discussion

A majority of participants completed all coaching sessions and outcome measures and feedback for CCS was positive. All participants stated they would recommend CCS, and a majority strongly agreed it was engaging and imparted useful skills. Participating in CCS was associated with significant increases in observer-rated overall communication skills, and there was no evidence indicating that it was associated with extended scan lengths. There were also significant increases in self-reported overall news delivery confidence ratings, in addition to significant confidence increases in all main forms of news delivery in obstetric ultrasound, including early pregnancy loss, late pregnancy/baby loss, early unexpected physical condition identification and late unexpected physical condition identification. There were no significant increases in the use of emotion words, or in either burnout subscale of exhaustion or disengagement. As it was a nominal variable, we did not conduct significance tests of the turnover intentions outcome variable. At baseline, all participants intended to stay in their job and only one sonographer changed their response at follow-up, indicating they were leaving for a similar job at another hospital. These findings extend the literature in three main ways.

First, this is the first time an evaluation of a tailored intervention to support sonographers with unexpected news delivery has been reported. Two previous evaluations of interventions to support news delivery in pregnancy have been conducted, but one of these focused on obstetricians [33] and another included a sample comprised mainly of midwives and nurses [34]. The immediate nature of news delivery in ultrasound presents unique challenges and as such, a tailored intervention which recognises and addresses these unique aspects is important. The present intervention was specifically tailored for this group. While the sample size was small and so our findings must be considered preliminary, the study identifies CCS as a promising candidate intervention with the potential for further evaluation in a larger, controlled study.

Second, this study presents the first observer-rating scale for sonographer communication skills. While a large number of studies have developed and used communication rating scales for news delivery by physicians [15] and for other forms of communication in a range of healthcare disciplines [35], no study has previously tried to capture and assess the communication practices of sonographers. Here, we present a rating scale based on consensus communication guidelines for sonographers, which captures change associated with participation in an intervention. There is a growing recognition of the need for sonographers to be trained in news delivery [8,36], and more organisations in a number of countries are adopting the practice of immediate news delivery by sonographers [6,8]. This rating scale could be a useful tool to appraise the communication skills of sonographers, identify their learning needs and evaluating the effectiveness of interventions.

Third, this is the first study to evaluate Communication Coaching for Sonographers (CCS). Previous studies have identified benefits of Communication Coaching in physicians, nurses and physician assistants in a range of healthcare settings [[18], [19], [20]], but this is the first to suggest Communication Coaching may also be useful in obstetric ultrasound. It is also the first evaluation of Communication Coaching for unexpected news delivery. Previous studies have suggested Communication Coaching can improve motivational interviewing skills, goals of care conversations and broader communication skills [[18], [19], [20],^25^]. This study further extends the evidence base by suggesting that Communication Coaching could help improve the way that potentially upsetting news is delivered to patients. In contrast to some previous studies, we found no evidence that Communication Coaching was associated with significant increases in emotion words [18,25]. However, this is likely to be due to our small sample size. Emotion naming was more than twice as frequent at follow up compared with baseline, but due to inter-participant variability, this did not reach significance. We also found no significant change in burnout, which contrasts with previous Communication Coaching studies [19,25]. An examination of means suggests this is unlikely to be attributable to a small sample size. Instead, due to Covid 19-related pressures on the UK healthcare system over the period the study was being conducted, it is possible that any benefits of a time-limited intervention may have been ‘cancelled out’ by work-related stressors.

While the present study focused on supporting communication in relation to unexpected news delivery, it must be acknowledged that sonographers can face a range of communication challenges in their work. Studies indicate that these are often due to a public misunderstanding of the purpose of obstetric scans, where these can be viewed as social occasions where patients ‘meet’ their baby, or learn their baby's gender, rather than as medical screening tests [37]. This misunderstanding can place sonographers in the difficult position of trying to undertake scans while simultaneously providing a pleasant social experience, in order to avoid receiving complaints about patients' experiences [38]. Future research should explore whether CCS may also have the potential to support sonographer communication in relation to these broader challenges.

The present study benefited from the inclusion of a varied range of outcome measures which were both self-reported and objectively measured. It also benefited from including an ethnically diverse sample, which reflects the population of sonographers the sample was drawn from. The study was limited by a small sample size, which means that findings must be interpreted with caution. Furthermore, there was no control arm, which means that any changes cannot be attributed solely to the intervention. Perhaps the most important limitation of the study was that we did not include patient-reported outcome data. This will be a crucial question to address in future studies, as the ultimate aim of the CCS is to improve the delivery of patient care. To address this, patients could be asked to rate their anxiety/distress following scans involving unexpected news, and this could be compared between sonographers who have received CCS and those who have not.

### 4.2 Innovation

Communication of obstetric findings by sonographers varies according to country and organisation [39]. In the UK, it has been standard practice for around 20 years and is now established in guidelines [16] and policy [8]. As such, the current findings are important for UK sonographers, who have been undertaking this challenging task without available evidence-based interventions for two decades. The current study addresses this gap by providing the first evaluation of a communication intervention for sonographers, which has the potential to be further evaluated and used in practice.

These findings will also have increasing relevance globally. Evidence clearly indicates that patients prefer to receive pregnancy ultrasound findings from the practitioner conducting the scan, and any delays in receiving unexpected news have a strong and deleterious impact upon their emotional wellbeing [3,12]. In response to this, immediate news delivery is being increasingly adopted as standard practice [6]. As such, we anticipate growing demand in coming years, internationally, for interventions such as CCS.

## 5 Conclusion

These findings demonstrate the potential usefulness of Communication Coaching for Sonographers conducting obstetric ultrasound scans. This could be important as the definition of ultrasound scans is increasingly improving, enabling more unexpected physical health conditions to be identified during pregnancy. However, they also have wider-reaching implications. Across healthcare settings, technological advances will continue to increase the speed with which a wide range of diagnostic results can be produced, for example, those relating to cancer and viral illnesses. Reduced delays are in patients' interests, but immediate diagnostic speeds will increase pressure on healthcare providers from a range of disciplines to communicate these findings clearly and sensitively, without the benefit of time to prepare. The current study positions Communication Coaching as a potentially useful intervention for supporting healthcare providers with immediate unexpected news delivery more broadly.

## Funding

This work was supported by the 10.13039/501100000862Sir Halley Stewart Trust [grant number: 24222, year: 2020].

## Declaration of Competing Interest

The authors have no competing interests to declare.
